# Microfluidic squeezing for intracellular antigen loading in polyclonal B-cells as cellular vaccines

**DOI:** 10.1038/srep10276

**Published:** 2015-05-22

**Authors:** Gregory Lee Szeto, Debra Van Egeren, Hermoon Worku, Armon Sharei, Brian Alejandro, Clara Park, Kirubel Frew, Mavis Brefo, Shirley Mao, Megan Heimann, Robert Langer, Klavs Jensen, Darrell J Irvine

**Affiliations:** 1 Department of Materials Science & Engineering, MIT; 2 Department of Biological Engineering, MIT; 3 David. H. Koch Institute for Integrative Cancer Research, MIT; 4 Department of Biology, MIT; 5 Department of Chemical Engineering, MIT; 6 The Ragon Institute of Harvard, MIT, and MGH; 7 Howard Hughes Medical Institute

## Abstract

B-cells are promising candidate autologous antigen-presenting cells (APCs) to prime antigen-specific T-cells both *in vitro* and *in vivo*. However to date, a significant barrier to utilizing B-cells as APCs is their low capacity for non-specific antigen uptake compared to “professional” APCs such as dendritic cells. Here we utilize a microfluidic device that employs many parallel channels to pass single cells through narrow constrictions in high throughput. This microscale “cell squeezing” process creates transient pores in the plasma membrane, enabling intracellular delivery of whole proteins from the surrounding medium into B-cells via mechano-poration. We demonstrate that both resting and activated B-cells process and present antigens delivered via mechano-poration exclusively to antigen-specific CD8^+^T-cells, and not CD4^+^T-cells. Squeezed B-cells primed and expanded large numbers of effector CD8^+^T-cells *in vitro* that produced effector cytokines critical to cytolytic function, including granzyme B and interferon-γ. Finally, antigen-loaded B-cells were also able to prime antigen-specific CD8^+^T-cells *in vivo* when adoptively transferred into mice. Altogether, these data demonstrate crucial proof-of-concept for mechano-poration as an enabling technology for B-cell antigen loading, priming of antigen-specific CD8^+^T-cells, and decoupling of antigen uptake from B-cell activation.

Antigen presenting cells (APCs) are a diverse subset of immune cells (including dendritic cells, macrophages, B-cells) that capture foreign or self proteins and peptides from tissues, and activate adaptive immune cells to generate either an inflammatory or tolerogenic immune response against these antigens. Proteins are ingested by APCs *in vivo* via fluid-phase sampling of their surroundings or receptor-mediated ingestion of foreign microbes or dead cell debris. Ingested proteins are degraded into peptide fragments (antigens) which are processed and presented to T-cells together with costimulatory signals, instructing naïve T-cell activation based on the specific signals received by the APC and the antigens presented. Because of this critical role in T-cell activation, purified APCs loaded with antigen and activated *ex vivo* can be used to expand functional T-cells in culture (e.g., for adoptive T-cell therapy) or as effective cellular vaccines *in vivo*. *Ex vivo* manipulation of APCs has gained increasing interest as an alternative approach for generating specific types of immunity, particularly cytotoxic T lymphocytes (CTLs) in diseases such as cancer^1^,^2^,^3^,^4^,^5^ and HIV^6^,^7^,^8^ where targeted killing of pathogenic cells is critical and endogenous APC function is actively suppressed. Despite promising preclinical studies, clinical translation of cell-based vaccines has been hampered by multiple limitations and only one APC-based vaccine is currently FDA-approved^9^,^10^.

Significant clinical research on cell-based vaccines has focused on dendritic cells (DCs), the so-called “professional” APCs because of their efficiency in priming CTLs, and their highly active extracellular protein uptake and antigen-processing capability. However, as a platform for clinical use, DCs are limited by their relative paucity in human blood^11^, complex subset heterogeneity^12^, short lifespan, and inability to proliferate. These challenges have led other cell types to also be considered for cell-based APC vaccines, including macrophages and B-cells^13^,^14^. In particular, B-cells have received interest for over a decade because of their unique properties as lymphocytes and their potential to overcome many limitations of DCs: B-cells are abundant in circulation (up to 0.5 million cells per mL of blood), can proliferate upon cellular activation, and efficiently home to secondary lymphoid organs when administered intravenously.

These potential advantages of B-cells as APCs are offset by limitations in the ability of B-cells to acquire and process antigen for priming of T-cells. B-cells express genetically rearranged B-cell receptors (BCR), which on binding to their target antigen, promote antigen uptake and B-cell activation. While B-cells are able to internalize antigens via their BCRs and prime primary T-cell responses^15^,^16^, their uptake of non-specific antigens (i.e. antigens not recognized by their BCR) is poor compared to macrophages and DCs, which efficiently pinocytose and phagocytose antigens from their surroundings. Furthermore, priming of CTLs occurs through presentation of peptide by class I MHC molecules, which are normally only loaded with antigens located in the cytosol (where the class I MHC processing machinery primarily resides). By contrast, proteins taken up via the BCR into endolysosomes tend to be directed to the MHC class II presentation pathway for presentation to CD4^+^T-cells^17^,^18^. Alternatively, B-cells and other professional APCs can load class I MHC molecules with peptides via cross presentation^19^,^20^,^21^,^22^,^23^,^24^, a process whereby class I peptide-MHC complexes are produced from endocytosed antigens via proteasomal processing or vacuolar protein degradation^25^, but this process is generally very inefficient.

Many methods have been developed to increase antigen uptake and cross-presentation in B-cells. These strategies largely rely on targeting specific receptors for endocytic uptake^16^,^20^,^26^, activating B-cells combined with fluid-phase protein exposure to increase non-specific endocytosis^16^, delivering antigen as immune-stimulating complexes^27^, or generating fusion proteins to direct B-cell function^28^. These approaches are limited by the fact that antigen uptake is coupled to other changes in B-cell state mediated by signalling through the targeted receptor, meaning that antigen loading and B-cell activation cannot be separately tuned. For example, resting B-cells have been shown to be tolerogenic to naïve CD8^+^T-cells, a potentially useful property in treating autoimmunity^29^,^30^, and activation of the B-cell would be problematic in such an application. Transfection of B-cells with DNA^31^,^32^, RNA^33^, or viral vectors^34^,^35^ encoding antigens has also shown promise, but is limited by a host of issues such as toxicity of electroporation, viral vector packaging capacity, transduction efficiency, stability, and anti-vector immunity.

Here, we demonstrate the application of a recently developed technology to facilitate direct cytosolic delivery of whole proteins into live B-cells by transient plasma membrane poration, induced as B-cells are passed through constrictions in microscale channels of a microfluidic device (mechano-poration)^24^,^36^. Using the well-defined model antigen ovalbumin (OVA), we demonstrate that delivery of whole protein via this method enables even resting B-cells to elicit robust priming of effector CTLs both *in vitro* and *in vivo*. To our knowledge, this novel method for whole protein delivery and antigen presentation by MHC class I is the first antigen delivery method in B-cells that easily decouples antigen loading from the process of B-cell activation, allowing these two processes to be separately tailored for immunogenic or tolerogenic vaccines. Cell squeezing provides an alternative modular platform that can prime autologous B-cells for *in vitro* CTL expansion as well as facilitate the development of B-cell-based vaccines.

## Methods

### Materials

TRITC- and Cascade Blue-labelled 3 kDa dextrans were purchased from Life Technologies. FITC-labelled 40 kDa dextran was purchased from Chondrex. Low endotoxin ovalbumin protein was purchased from Worthington Biochemical Corporation. CpG ODN 1826 (CpG B), CpG ODN 2395 (CpG C), and LPS *Escherichia coli K12* (LPS) were all purchased from Invivogen. Multimeric/megaCD40L was purchased from Adipogen and Enzo Life Sciences.

### Mice

All procedures were performed under an animal protocol approved by the Massachusetts Institute of Technology Committee on Animal Care (CAC) and in accordance with the guidelines for animal care in a Massachusetts Institute of Technology animal facility inspected by the US Department of Agriculture. Animals were cared for in the USDA-inspected MIT Animal Facility under federal, state, local and NIH guidelines for animal care. C57BL/6J (B6) mice were purchased from Jackson Labs. CD45.1 B6, OT-I Thy1.1, and OT-II strains were maintained in-house, with periodic genotyping from Transnetyx. All mice used were female and 6–12 wks of age.

### Cell isolation

For B-cell isolation, spleens were harvested from mice and mashed through a 70 μm cell strainer. Red blood cells were lysed and resting B-cells were isolated from the cell suspension with the B-Cell Isolation Kit, mouse (Miltenyi Biotec) following the manufacturer’s instructions. After isolation, B-cell suspensions were>95% B220+, as measured by flow cytometry. For CD8^+^T-cell isolation, spleens and inguinal lymph nodes were harvested and mashed. Following red blood cell lysis, CD8a^+^T-cells were isolated with the CD8a^+^T-cell Isolation Kit, mouse (Millenyi Biotec) according to the manufacturer’s instructions. CD4^+^T-cell isolation was performed with the CD4^+^T-cell Isolation Kit, mouse (Millenyi Biotec) on spleen and inguinal lymph node suspensions. T-cells were consistently >90% pure, as measured by CD8a or CD4 staining and flow cytometry. All cell culture was performed in T-cell media (RPMI with 10% FBS, penicillin-streptomycin, 1X sodium pyruvate) supplemented with 1 μL/mL of 55 mM β-mercaptoethanol.

### Protein delivery by cell squeezing

Delivery of whole protein antigen to resting B-cells was performed using CellSqueeze, a commercial microfluidics device and pressure system (SQZ Biotech); chip designs used included 30-4 × 1, 10-4 × 1, and 30-5 × 5 where X-YxZ denotes Z sequential constriction channels of dimensions Xμm long and Yμm diameter. B-cells were suspended at 5 × 10^6^ cells/mL in media with 100 μg/mL of ovalbumin, 0.3 mg/mL TRITC- or Pacific Blue-labelled 3 kDa dextran, or 0.3 mg/mL FITC-labelled 40 kDa dextran, and placed on ice. The microfluidics chips and holder set were also placed in an ice water bath until cold. The cell suspension was sent through the device in 200 μL aliquots at 120 psi. Endocytosis control B-cells were prepared identically in medium with OVA, but did not go through the microfluidics device. After antigen loading, cells were allowed to rest at room temperature for 5 minutes and washed twice with PBS. To assess delivery efficiency, uptake of fluorescently-labelled dextrans was measured by flow cytometry as described below.

### *In vitro* cell culture, activation & proliferation assays

To characterize *in vitro* activation of mechano-porated B-cells, cells that went through the SQZ device and endocytosis control cells were incubated in a 96-well U-bottom plate at 5 × 10^5^ cells/mL with 5 μM CpG B, 5 μM CpG C, or 100 ng/mL LPS. Flow cytometry was performed at 24 and 48 hours to measure cell-surface levels of CD86, CD40, CD69, MHC class I, and MHC class II as described below.

For *in vitro* proliferation assays, purified OT-I CD8^+^ or OT-II CD4^+^T-cells were suspended at 10^7^ cells/mL and labelled with CFSE (5 μM, Life Technologies) for 10 min. After one wash, T-cells and B-cells were plated at a 1:0.8 ratio in 200 μL of T-cell media in a 96-well U-bottom plate. CpG B, CpG C, or LPS was added to the appropriate wells, and anti-CD3/CD28 Dynabeads (Life Technologies) were added to positive control wells at 1 bead/T-cell. Supernatant collection for cytokine analysis and flow cytometry to assess T-cell proliferation were performed on day 2 and day 4.

### Luminex cytokine assay

Supernatants were collected and cleared of cells by centrifugation on days 2 and 4 of *in vitro* proliferation assays. Cytokine concentrations were measured using a custom Milliplex MAP Mouse Cytokine/Chemokine Magnetic Bead assay (Millipore) following the manufacturer’s protocol with the following modifications: capture beads and detection antibodies were used at a 1/5x recommended dilution. Assays were read on a Bio-plex FLEXMAP 3D (Bio-Rad).

### *In vivo* proliferation assay

On day -1, 10^6^ OT-I Thy1.1 CD8^+^resting T-cells labelled with CFSE (5 μM) were injected retro-orbitally (r.o.) into C57BL/6 mice. The next day (day 0), animals were injected r.o. with 1–3 million CD45.1^+^B-cells that had been loaded with OVA using the microfluidics SQZ device the previous day and incubated overnight with 5 μM CpG B, or were loaded with OVA by mechano-poration just prior to injection and not exposed to any TLR ligand. Animals were necropsied on day 4 and their spleens, inguinal lymph nodes, and cervical lymph nodes were harvested. The organs were mashed through a cell strainer. Single-cell suspensions were incubated with mouse anti-CD16/CD32 (eBioscience) to reduce nonspecific antibody binding, and were stained with anti-CD8-APC, anti-B220-PE-Cy7, anti-CD45.1-PerCP-Cy5.5, and anti-Thy1.1-APC-Cy7. Flow cytometry to determine cell numbers and Thy1.1+CD8^+^T-cell CFSE dilution was performed.

### Analysis of proliferation data

Normalized cell counts were calculated by dividing the number of surviving T-cells sampled in an experimental condition by the average number of surviving T-cells in the T-cell only control from the same experiment and assay timepoint. To calculate percent of input cells undergoing division and proliferation index, T-cells were first gated into generations according to their levels of CFSE dilution. The live T-cell count for each generation was divided by 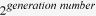 to calculate the number of original cells that had divided to create the cells in that generation gate. The percent of original cells divided was calculated by dividing the number of original cells that had divided by the total number of original cells. The proliferation index quantifies the mean number of divisions each divided cell underwent, and is given by 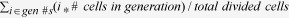.

### Flow cytometry

Cell populations were identified with anti-B220 (CD45R)-PE-Cy7 (RA3-6B2, eBioscience), anti-CD8a-APC (53-6.7, eBioscience), anti-CD4-AF647 (GK1.5, BioLegend), anti-CD45.1-PerCP-Cy5.5 (A20, BioLegend), and anti-Thy1.1 (CD90.1)-APC-Cy7 (OX-7, BioLegend). Cell surface activation marker levels were assessed by staining with anti-CD86-BV650 (GL-1, BioLegend), anti-CD40-APC (1C10, eBioscience), anti-CD69-PerCP-Cy5.5 (H1.2F3, BioLegend), anti-MHC I (H-2Kb)-PE (AF6-88.5.5.3, eBioscience), and anti-MHC II (I-A/I-E)-FITC (M5/114.15.2, eBioscience). All antibodies were used at a 1:500 dilution. Live-dead staining was performed with DAPI (3 μM wash), or propidium iodide (PI; 1:50 dilution). Data collection was performed on a BD LSRFortessa, BD FACSCanto, or BD LSR II cytometer.

### Statistical analyses

One- or two-way analysis of variance (ANOVA) was performed between treatment groups on normalized data. Holm Sidak multiple comparisons post-tests were performed, relevant comparisons were selected, and multiplicity adjusted P-values were reported. P-values less than or equal to 0.05 were considered statistically significant. All statistical analyses were performed with Prism 6.04 software (GraphPad).

## Results

### Mechano-poration enables rapid delivery of macromolecules to resting, polyclonal B-cells without altering function

**Figure 1:**
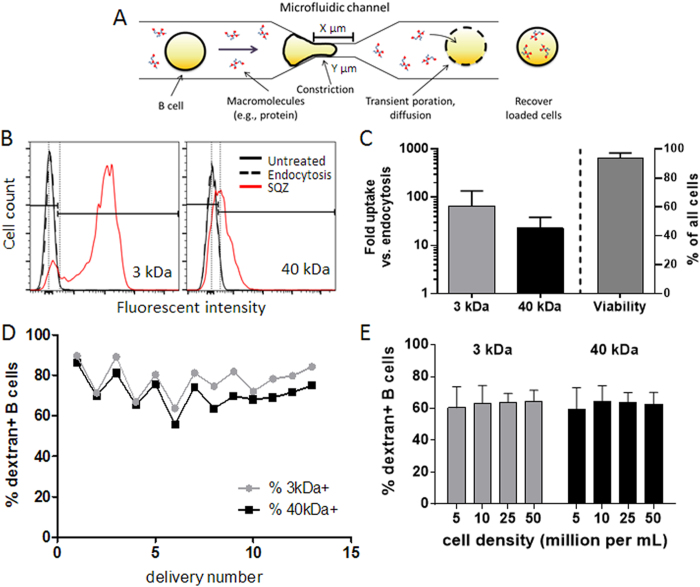
Microfluidic cell squeezing provides a robust, scalable method for macromolecule delivery to B-cells. **A)** Schematic representation of microfluidic squeezing for macromolecule delivery to cells. Constriction channels were X μm long (10 or 30 μm) and Y μm in diameter (4 or 5 μm); these values were 30 μm long and 4 μm in diameter unless otherwise indicated. **B)** Representative histograms and gating strategy for assessing device performance for delivery of small (3 kDa) and large (40 kDa) dextrans in B-cells under these antigen delivery conditions: untreated; endocytosis; SQZ=squeezed (mechano-poration). **C)** Quantitative analysis of macromolecule uptake into resting B-cells relative to endocytosis; cell viability following mechano-poration (*n* = 7 independent experiments). **D)** Quantitative analysis of intra-device delivery performance (*n* = 13 consecutive runs using 1 device; results representative of >3 independent experiments). **E)** Dextran delivery into resting B-cells as a function of cell density from 5-50 million B-cells/mL (*n* = 3 replicates; representative of>3 independent experiments). All data were represented as means±standard deviation. Pairs of conditions were tested in **E)** for statistically significant differences with ordinary 1-way ANOVA followed by Holm Sidak multiple comparisons test; multiplicity adjusted p-values < 0.05 were considered significant, but none were found.

The process of mechano-poration for loading B-cells with antigen is illustrated in Fig. 1A. Live cells are passed through parallel microfluidic channels in a silicon device; in each channel, 1 or more constrictions create transient pores in the membranes of cells passing through the device. Macromolecular cargos present in the surrounding fluid can diffuse into the cell during this transient poration, leading to intracellular loading. Previously, we demonstrated that mechano-poration is effective for promoting cytosolic delivery of macromolecules into a wide variety of cell types including primary murine B-cells^24^; efficient delivery was achieved with 5 sequential constrictions with dimensions 30 μm length and 5 μm width. However, recent iterations in device design (increased number of parallel constriction channels to 75, longer entry region, reversibility, etc.)^36^ prompted us to revisit optimal mechano-poration parameters for protein delivery into B-cells and subsequent antigen presentation. Pilot optimization experiments showed that 30-4 × 1 microfluidics chips (1 constriction per channel that is 30 μm long and 4 μm in diameter) run at 120 psi were the most effective chip design for mechano-poration of murine B-cells with both efficient delivery and high cell viability at concentrations of 5 × 10^6^ B-cells/mL (Supplementary Fig. S1A shows representative delivery of 30-5 × 5 vs. 30-4 × 1); these conditions were used for all subsequent delivery experiments. Cells at this pressure ran through a device at a rate of approximately 1 million cells per second. We performed a quantitative assessment of inter-device and intra-device variability in process efficacy for resting polyclonal murine B-cells, and compared the uptake of low and high molecular weight dextrans (3 and 40 kDa, respectively) delivered into B-cells via mechano-poration (condition abbreviated as “SQZ” in the figures). In parallel, control B-cells were incubated with the same polymers for the duration of mechano-poration (~30 min) to compare levels of internalization achieved by B-cells through steady-state fluid-phase endocytosis/pinocytosis (“endocytosis”). Representative histograms of dextran uptake by B-cells following mechano-poration (“SQZ”) vs. controls are shown in Fig. 1B. As shown in Fig. 1C (left), microfluidic squeezing promoted greatly enhanced dextran uptake compared to endocytosis, with internalization increased ~65-fold and ~25-fold for 3 and 40 kDa dextrans, respectively. This represented delivery to 75-90% of all cells for both dextrans; by comparison less than 10% of resting B-cells endocytosed detectable amounts of cargo (Fig. 1B,D). Viability of recovered cells after mechano-poration was ~95% (Fig. 1C; right), similar to endocytosis controls. The manufacturer-specified capacity for the maximum number of cells that can be passed through these disposable microfluidic chips ranged from 1-5 million cells per device; however, here devices were run with multiple aliquots of cells (1 million cells per run) until clogged, and the maximum number of cells run through each individual device in a given experiment was often in significant excess of 5 million cells. Importantly, we determined there was low variability in percentage of delivered cells from first run to clogging of devices, indicating that intra-device variability was minimal (Fig. 1D). We also found that delivery performance of multiple devices within the same experimental session (inter-device variability) was very consistent (Supplementary Fig. S1B). Recognizing that some applications may require higher numbers of B-cells, we also tested the efficacy of the microfluidic devices at different cell densities. The efficiency of intracellular dextran delivery was largely independent from cell concentration up to at least 50 × 10^6^ cells per mL, indicating potential for robust scalability (Fig. 1E).

**Figure 2:**
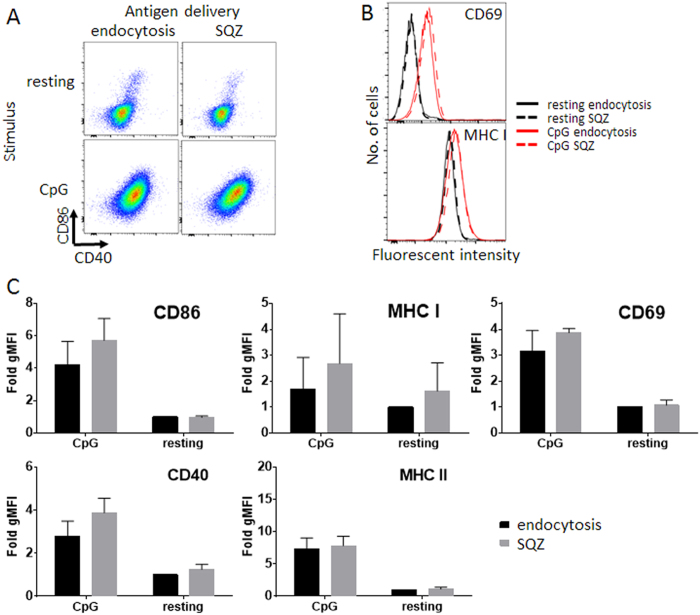
B-cell activation are not altered by cell squeezing. Representative pseudocolor 2D plots (**A CD40 vs. CD86**) and histograms (**B CD69 and MHC I**) from flow cytometry for activation and antigen presentation markers of resting or CpG-stimulated B-cells passed through the mechano-poration chip or incubated for an equivalent period in media (endocytosis). **C)** Quantitative analysis of activation and antigen-presentation marker expression after 48 h in media alone (resting) or CpG B 1826 stimulation (CpG), calculated as fold change in geometric mean fluorescence intensity above resting, endocytosis cells. All data were represented as means±standard deviation (*n ≥ *3 independent experiments). Pairs of conditions were tested in **C)** for statistically significant differences between SQZ and endocytosis with ordinary 1-way ANOVA followed by Holm Sidak multiple comparisons test; multiplicity adjusted p-values < 0.05 were considered significant, but none were found.

To be effective as APCs for priming effector T-cells, B-cells need to be both loaded with antigen and activated, as activation upregulates antigen presentation and the expression of costimulatory receptors required for naïve T-cell activation^16^. By contrast, for promotion of tolerance, un-activated or alternatively activated B-cells loaded with antigen may be desirable. Thus, we next assessed whether the process of mechano-poration impacts B-cell activation, in the presence or absence of defined B-cell stimuli. CpG DNA is a ligand for Toll-like receptor (TLR)-9 that has been extensively explored as a vaccine adjuvant and B-cell stimulus that enhances antigen uptake and activates both mouse and human cells^20^,^37^,^38^. Using CpG as a model activation stimulus, we longitudinally phenotyped multiple surface markers of B-cell activation and antigen presentation to evaluate (i) whether resting B-cells were activated by mechano-poration and (ii) whether mechano-poration altered the activation of CpG-stimulated B-cells. As shown in Figs. 2A-C, microfluidic squeezing did not significantly induce the expression of activation (CD69), co-stimulation (CD40, CD86), or antigen presentation (MHC class I, II) markers in resting B-cells. Mechano-poration also did not alter the upregulation of these markers induced by treatment with CpG for 48 h (Fig. 2A-C). Thus, alterations induced by the mechanical stress of cell squeezing were non-stimulatory for resting B-cells, and squeezed B-cells maintained normal functional responses to stimulation. This also held true for other candidate B-cell stimuli including class C CpG oligonucleotides, LPS, and multimeric CD40L (Supplementary Fig. 2).

### Polyclonal B-cells squeezed with whole protein expand antigen-specific CD8^+^T-cells that secrete effector cytokines *in vitro*

**Figure 3:**
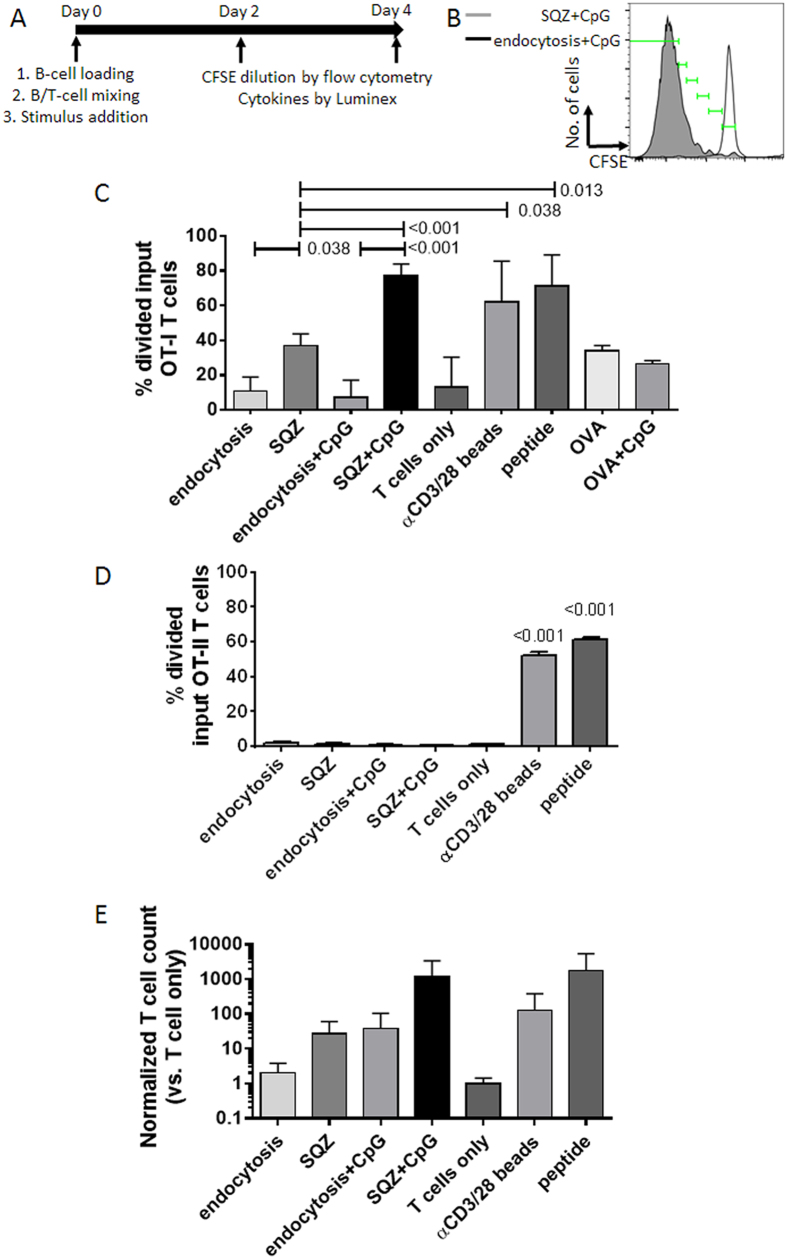
Whole protein delivery to B-cells by cell squeezing enables robust MHC class I antigen presentation and antigen-specific CD8^+^T-cell priming *in vitro*. **A)** Experimental timeline for antigen loading (endocytosis, peptide, or squeezed) on day 0 followed by co-culture with purified, CFSE-labelled OT-I CD8^+^T-cells. **B**) Representative histogram overlay showing data from day 4 proliferation of endocytosis+CpG or SQZ+CpG co-cultures; green gates were used to calculate percent of divided input cells and proliferation indices as described in Methods. **C,D**) Quantitative analysis of percent divided input OT-I CD8^+^(**C**) and OT-II CD4^+^(**D**) T-cells at day 4 of co-culture. **E**) Normalized total OT-I CD8^+^T-cell counts on day 4 of co-cultures were also calculated as described in Methods. All data were shown as means±standard deviation (*n* = 7 independent experiments for **B** & **D***; n = *3 independent experiments for **D**). Pairs of conditions were tested in **C**), **D**), and **E**) for statistically significant pairwise differences with ordinary 1-way ANOVA followed by Holm Sidak multiple comparisons test; multiplicity adjusted p-values < 0.05 were considered significant, and exact *p*-values were shown where significant. All conditions in **D**) were significantly lower than positive controls (αCD3/28 beads, peptide).

To determine whether mechano-poration can facilitate protein delivery to the cytosolic class I MHC antigen processing and presentation machinery, we utilized the optimal conditions described above to deliver the model protein ovalbumin (OVA) into resting, purified polyclonal B-cells, by passing cells through microfluidic device in the presence of excess OVA in the surrounding medium. Squeezed B-cells were then co-cultured with CFSE-labelled OT-I CD8^+^ T-cells, which bear a transgenic T-cell receptor specific for the MHC class I-restricted OVA peptide SIINFEKL, in the presence or absence of CpG as a B-cell-activating stimulus (Fig. 3A). CFSE dilution in OT-I T-cells was analysed by flow cytometry to assess proliferation/expansion of the T-cells in response to B-cell-presented antigen (gating strategy presented in Fig. 3B & Supplementary Fig. S3). As positive controls, T-cells were cultured with anti-CD3/CD28-coated beads or with B-cells incubated continuously with the optimal SIINFEKL peptide; T-cells were cultured alone with no stimulation as negative controls.

B-cells incubated with OVA in solution to allow antigen uptake by endocytosis for the same duration as microfluidic squeezing (30 min) elicited no T-cell proliferation above background, regardless of whether CpG was subsequently added to activate the B-cells in co-culture (endocytosis, endocytosis+CpG, Fig. 3C). This weak response was only modestly enhanced by continuous incubation of the B/T co-cultures for 4 days with high concentrations of soluble OVA (100 μg/mL); ~30% of the input CD8^+^T-cells divided with or without B-cell activation by CpG in these conditions (Fig. 3C, OVA and OVA+CpG, respectively). These results are consistent with the low efficiency of endocytic antigen uptake and cross presentation by B-cells. Notably, prior studies have also shown that when APCs are continuously exposed to antigen and CpG, antigen uptake and cross presentation to T-cells is very limited unless the antigen and CpG are directly conjugated^20^,^37^. By contrast, CD8^+^T-cells incubated with squeezed B-cells proliferated significantly in both resting and activated co-cultures; co-cultures that were resting or continuously activated by soluble CpG had approximately 40% and 80% input CD8^+^T-cells undergoing division, respectively (Fig. 3C, p = .038 and < 0.001 vs. endocytosis for resting and CpG, respectively). CpG-activated squeezed B-cells stimulated the majority of T-cells to divide by 4 days of co-culture, essentially identical to positive controls of T-cells primed by anti-CD3/CD28-coated beads or B-cells loaded with saturating amounts of SIINFEKL peptide (Fig. 3C). This result is striking given the relative inefficiency of cytosolic antigen processing, and suggests that squeezing led to high levels of antigen uptake that may have saturated MHC class I loading with the target peptide. However, cell squeezing should primarily direct antigen to the cytosol and not endosomal compartments where MHC class II loading occurs. Consistent with this, we found neither resting nor CpG-activated mechano-porated B-cells were able to expand OVA-specific OT-II CD4^+^T-cells after 4 days of co-culture (Fig. 3D).

We also assessed proliferation index (the average number of divisions for all dividing cells) and counts of recovered T-cells to determine survival of proliferating OT-I cells primed by mechano-porated B-cells. Mechano-porated, activated B-cells drove T-cells to undergo progressive division for an average of ≥3 generations, equivalent to the anti-CD3/CD28 bead and SIINFEKL peptide positive controls (Supplementary Fig. S4). T-cell counts at day 4 showed that CpG-activated squeezed B-cells promoted the best expansion of T-cells by far among the tested groups, yielding 10-fold more OT-I cells than activated B-cells loaded with antigen by endocytosis or resting B-cells loaded by squeezing (Fig. 3E). Further, more T-cells were recovered from activated/squeezed B-cell co-cultures than cultures of T-cells primed with anti-CD3/28-coated beads (a first-line platform for T-cell expansion in adoptive therapy clinical trials^39^), and this group matched the T-cell expansion achieved by B-cells loaded by continuous optimal peptide exposure (Fig. 3E).

**Figure 4:**
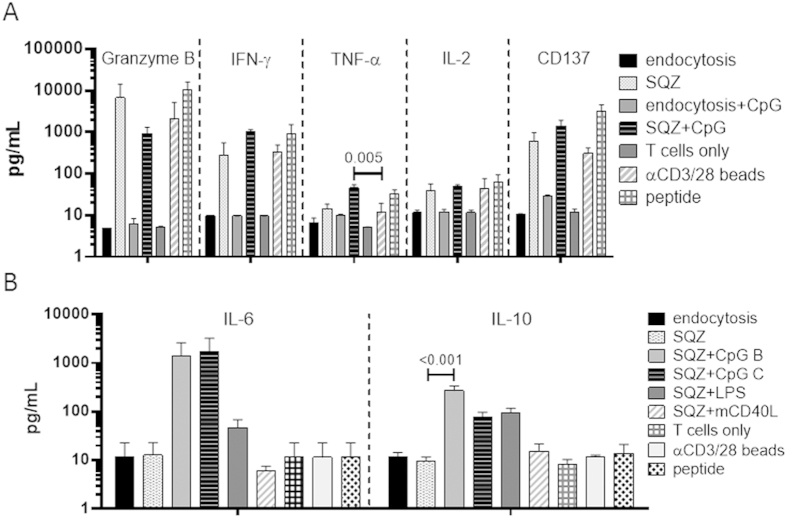
Squeeze-delivered B-cells prime T-cells to produce effector CTL cytokines, while B-cell stimuli program distinct cytokine milieus. **A)** Secretion of major effector cytokines and molecules (granzyme B, IFN-γ, TNF-α, IL-2, CD137) were shown for co-cultures indicating T-cell activation state. **B**) Differential IL-6/IL-10 milieus induced in co-cultures by activating with different B-cell stimuli (*n* = 3 independent experiments for **A** & **B**). All data were represented as means±standard deviation. Pairs of conditions were tested for statistically significant pairwise differences with repeated measures 1-way ANOVA followed by Holm Sidak multiple comparisons test; multiplicity adjusted p-values < 0.05 were considered significant, and exact *p*-values were shown where significant.

The functionality of B-cell-primed CD8^+^ T-cell populations was assayed by measuring secretion of effector molecules at days 2 and 4 of co-cultures. B-cells loaded with antigen by squeezing, whether resting or activated, primed T-cells to secrete substantial quantities of granzyme B, IFN-γ, and TNF-α, while B-cells loaded with antigen by endocytosis produced basal levels of cytokines (Fig. 4A). CpG-activated, squeezed B-cells primed CD8^+^T-cells that produced less granzyme B, but more IFN-γ and TNF-α when compared to resting squeezed B-cells, though these differences did not reach statistical significance. IL-2 and activation-induced release of soluble CD137 were also detected^40^, consistent with T-cell activation by both resting and activated mechano-porated B-cells (Fig. 4A). Effector cytokine secretion levels were similar between squeezed B-cells and positive controls--beads or peptide. In addition to T-cell-derived cytokines, we also examined potential cytokines in the co-culture milieu that could direct CTL priming and output. Different B-cell stimuli produced distinct cytokine secretion patterns as expected: CpG- and LPS-stimulated cells generated divergent ratios of IL-6 and IL-10 secretion, while mCD40L induced low levels of both (Fig. 4B). Thus, B-cells loaded with antigen by mechano-poration primed functional effector T-cells and produced an IL-6/IL-10 milieu that is known to be important in programming different priming, expansion, and differentiation potentials for effector^41^,^42^,^43^,^44^ and memory CD8^+^T-cells^45^,^46^. Consistent with previous studies, combined IL-6 and IL-10 expression correlated with increased proliferation indices in innate-stimulated (CpG B, CpG C, LPS) co-cultures, while resting (SQZ) co-cultures had relatively lower proliferation and produced negligible IL-6/IL-10 (Supplementary Fig. S4).

### Squeezed B-cells prime antigen-specific CD8^+^T-cells *in vivo*

**Figure 5:**
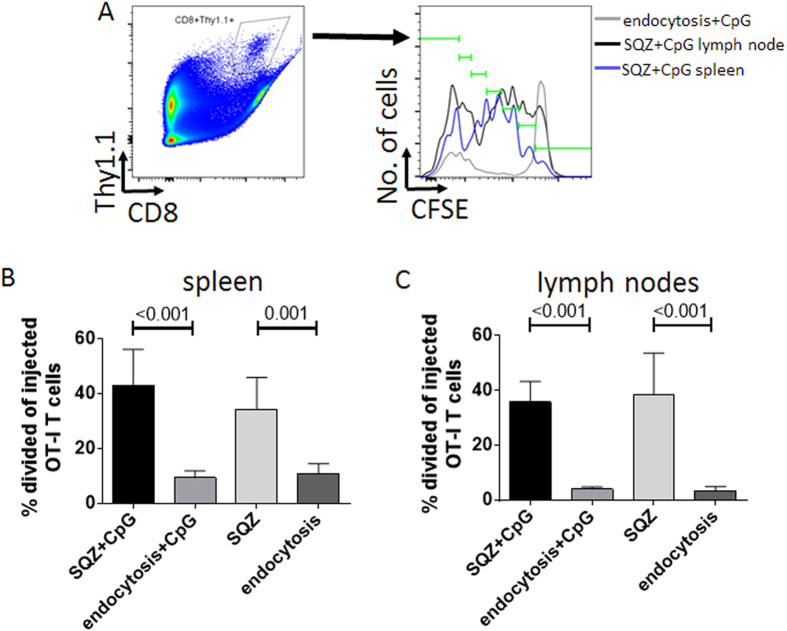
Antigen-specific CD8^+^T-cell proliferation is primed *in vivo* by squeeze-delivered B-cells. **A)** Representative gating shown on 2D pseudocolor plot (CD8 vs. Thy1.1) to identify adoptively transferred OT-I T-cells, followed by representative histogram overlays of CFSE dilution and gating to identify number of divisions. **B**, **C)** Quantitative analysis shown for % divided OT-I T-cells in spleens (**B**) and inguinal lymph nodes (**C**). All data are represented as means±standard deviation (*n* = 3 animals, representative of 3 independent experiments). Pairs of conditions were tested for statistically significant differences with ordinary 1-way ANOVA followed by Holm Sidak multiple comparisons test; multiplicity adjusted p-values < 0.05 were considered significant and exact *p*-values were shown where significant.

In addition to having potential as an *in vitro* antigen-specific T-cell expansion platform, B-cells are an alternative to dendritic cells for use as cellular vaccines. As proof-of-principle for this application, we adoptively transferred CFSE-labelled OT-I CD8^+^T-cells expressing Thy1.1 as a congenic marker into recipient mice as reporters of antigen presentation. One day later, we injected either resting B-cells immediately after mechano-poration-mediated antigen loading, or mechano-poration-loaded B-cells that were subsequently activated for 24 h with CpG *in vitro*. Resting B-cells loaded with antigen by endocytosis were used as controls, either immediately or after 24 h of activation with CpG. Four days after B-cell transfer, mice were sacrificed and spleens and inguinal lymph nodes were analysed for OT-I proliferation by flow cytometry. Representative results and flow cytometry gating are shown in Fig. 5A and Supplementary Fig. S5. Consistent with our *in vitro* results, we found that mechano-porated B-cells were able to elicit division of adoptively transferred OT-I T-cells while endocytosis controls showed only basal division. Both CpG-activated and resting squeezed B-cells caused OT-I proliferation in spleens (Fig. 5B, ~45% and ~35% divided of injected OT-I T-cells with p < 0.001 and p = 0.001 comparing SQZ vs. endocytosis, respectively). CpG-activated and resting squeezed B-cells also elicited similarly enhanced OT-I proliferation in lymph nodes compared to endocytosis B-cells (Fig. 5C, ~40% and ~35% divided of injected OT-I T-cells by CpG B and resting squeezed B-cells, respectively; p < 0.001 comparing SQZ vs. endocytosis for both resting and CpG). Endocytosis controls showed ~4% baseline division in lymph nodes. These promising results suggested that B-cells loaded by microfluidic mechano-poration merit further investigation as an *in vivo* platform for cellular vaccines.

## Discussion

B-cells loaded with specific antigens have been proposed as candidate APCs for cell-based vaccines and as autologous reagents for expansion of antigen-specific T-cells, with potential advantages over dendritic cells in these applications, especially with respect to their ready availability in large numbers from peripheral blood and their ability to be further expanded substantially in culture. However, methods for efficient antigen loading in B-cells, especially for class I MHC presentation, have been a major barrier to development of polyclonal B-cells as APCs for *in vitro* or *in vivo* use. Here we have demonstrated a simple approach using microfluidic-based mechanical deformation and passive diffusion that enables robust loading of protein antigens directly to the cytosol of resting or activated B-cells, leading to MHC class I presentation of peptides derived from native whole protein. The advantages conferred by mechano-poration are numerous, including the potential to deliver native proteins without processing, engineering, or modification, the rapid nature of the process, the relatively high efficiency of delivery and functional outcomes, and the ability to deliver material to resting cells decoupled from cellular biology such as programming stimulus or receptor targeting. Alternate strategies for direct protein loading have been attempted, including electroporation, with limited success in standard bulk formats, requiring engineered, highly controlled electrical fields^47^,^48^. These physical strategies also rely on application of energy and forces that may damage proteins, whereas for the fluidic strategy used here, extraordinarily high shear flow is required for theoretical protein denaturation and has not been observed^49^. Most clinical strategies for *ex vivo* T-cell expansion for adoptive transfer rely on extensive activation and pulsing with tumour lysate or peptides using large numbers of PBMC feeder cells, APCs or αCD3/28-coated beads^50^,^51^,^52^. Our data suggest that mechano-porated B-cells may be a useful alternative source for autologous, antigen-loaded APCs that can either match or exceed performance in CTL expansion and effector function compared to other platforms such as beads or peptide pulsing. Further, *in vivo* priming of antigen-specific T-cells by mechano-poration loaded B-cells suggests novel opportunities for combined cellular immunotherapies similar to previous clinical strategies pairing genetically engineered T-cells with *ex vivo* prepared APCs^50^,^53^.

The ability to deliver whole native protein or polypeptides directly to the cytosol for processing and MHC class I presentation enables unbiased presentation of multiple peptides following native antigen processing. The microfluidic cell squeezing device should also enable delivery of mixtures of proteins, including tumour lysates or other complex protein sources in future applications. This would greatly facilitate progression of fields such as personalized vaccines by facilitating loading of tumour biopsy lysates. Without the need for extensive genetic engineering, protein engineering, biochemical modifications, and viral or synthetic packaging of protein, mechano-poration facilitates the use of a wide range of targets with fewer concerns about complications such as insert size, charge interactions etc. Aside from this study’s focus on proteins, squeezing as a platform is readily multiplexed with other cargoes, providing access to most existing technologies as previously demonstrated by intracellular delivery of siRNA, nanoparticles, antibodies, and transcription factors^24^. Our demonstration that proteins can be delivered for MHC class I processing independently of cell activation status is a major advantage of this approach.

To this point, APC activation stimuli are generally constrained by the requirement that they must enable antigen uptake. Our approach enables decoupling of protein loading and cellular programming, facilitating independent modulation of each component. This could have broad implications for the types of vaccines that can be programmed, such as enabling MHC class I presentation in cytokine environments that do not favour endocytic uptake or cross-presentation. Our results of OT-I proliferation, and in particular effector cytokine secretion primed by resting squeezed B-cells are interesting considering the well-documented tolerogenic potential of resting B-cells^29^,^30^. Tolerised T-cells exist along a functional spectrum including cells that can proliferate in response to initial priming, but are unable to secrete cytokines or to respond extensively to subsequent antigen exposures as seen in chronic viral infections^54^, CD3/CD28 re-stimulation *in vitro* without help^55^, and tolerance^56^; future studies should focus on elucidating how mechano-poration B-primed T-cells function in light of previous studies. This system may enable unique basic biology investigations by increasing the modularity of cellular activation and antigenic protein compartmentalization, and allowing antigen loading without introducing confounding biological effects on the cell such as may occur when using a viral or other biological vector to introduce antigen into APCs. For example, our data indicating divergent IL-6/IL-10 cytokine milieus produced by different innate and adaptive immune B-cell stimuli can allow investigation of the biology of these pleiotropic molecules produced by B-cell APCs independent of their effects on B-cell antigen uptake and MHC class I processing/presentation.

Although cellular mechano-poration has demonstrated much potential, the current iteration of the technology has certain technical limitations. For delivery of larger macromolecular cargos, there is a trade-off between more aggressive mechano-poration for increased delivery and decreased cell viability/increased potential device clogging. Such challenges can potentially be overcome through the development of optimized constriction designs and increasing molar concentration of cargo in solution to drive higher diffusion rates. The current device design contains 75 parallel constriction channels, limiting the maximum number of cells processed per device run; future increases in the number of channels or operating multiple devices in parallel can dramatically improve throughput. The ease of use and rapid processing enabled by our approach (~1 × 10^6^ cells/s rate flow through device,<2 h for total experiment time) can provide a host of benefits for clinical translation. Perhaps most obvious is the reduction in time and resources required for preparation of cell-based therapeutics. One prospective implementation would be a true bedside preparation process using a self-contained, sterile, consumable device; however, the current iteration includes a large external pressure system. The development of low pressure chip designs could make a bedside implementation more feasible by creating a smaller fluid control footprint. In addition to current technological limitations, one potential biological limitation may be the absence of MHC class II presentation and priming of CD4^+^T-cells. CD4^+^T-cell help can contribute as an important component in developing CD8^+^T-cell responses, particularly supporting the recall of memory^57^,^58^,^59^, which was not directly tested in our proof-of-concept studies. Future studies might address this limitation simply by complementing mechano-poration with direct surface MHC II loading of T-helper peptides to generate CD4^+^T-cell help. Alternatively, other modifications and cell stimuli could be utilized to enhance MHC class II processing of proteins delivered via mechano-poration.

In our application of B-cells as APCs, the amount of patient blood required to prepare a single-dose cellular vaccine would be vastly reduced compared to DC-based approaches, and we can successfully avoid time required for protein loading by cellular uptake processes such as endocytosis or pinocytosis. Our results *in vitro* demonstrated significant potential for B-APCs as an alternative platform for expansion of effector CTLs. These squeezed B-cells also functioned as effective APCs *in vivo*, though many factors remain to be optimized here such as ideal B-cell activation stimulus and timing, and establishing methods to co-load both MHC class I and class II antigen presentation pathways to generate CD4^+^T-cell help. Future studies should be designed to address these potential limitations, which are concerns for many B-cell-based vaccine approaches.

## Author Contributions

G.L.S. and D.J.I. conceived the study and designed experiments. G.L.S., D.V.E., H.W., B.A., C.P. and K.F. performed and optimized experiments, analysed data, wrote the manuscript, and prepared figures. M.B., S.M. and M.H. performed experiments. S.M., M.H., A.S., R.L. and K.J. contributed reagents and expertise for optimizing devices and SQZ system support. All authors critically revised and approved of the manuscript.

## Additional Information

**How to cite this article**: Lee Szeto, G. *et al.* Microfluidic squeezing for intracellular antigen loading in polyclonal B-cells as cellular vaccines. *Sci. Rep.* **5**, 10276; doi: 10.1038/srep10276 (2015).

## Supplementary Material

Supplementary Information
