# Supplementary material for: Microfluidic squeezing for intracellular antigen loading in polyclonal B-cells as cellular vaccines
Source: Sci Rep. 2015 May 22;5:10276. doi: 10.1038/srep10276 (PMC4441198; doi:10.1038/srep10276)
Supplement: Supplementary Information [file srep10276-s1.pdf]

Supplementary Information

Microfluidic squeezing for intracellular antigen loading in polyclonal B-cells as cellular vaccines

Authors: Gregory Lee Szeto, Debra Van Egeren, Hermoon Worku, Armon Sharei, Brian Alejandro, Clara Park, Kirubel Frew, Mavis Brefo, Shirley Mao, Megan Heimann, Robert Langer, Klavs Jensen, Darrell J Irvine*

This file includes:

Supplementary Figures S1 to S5

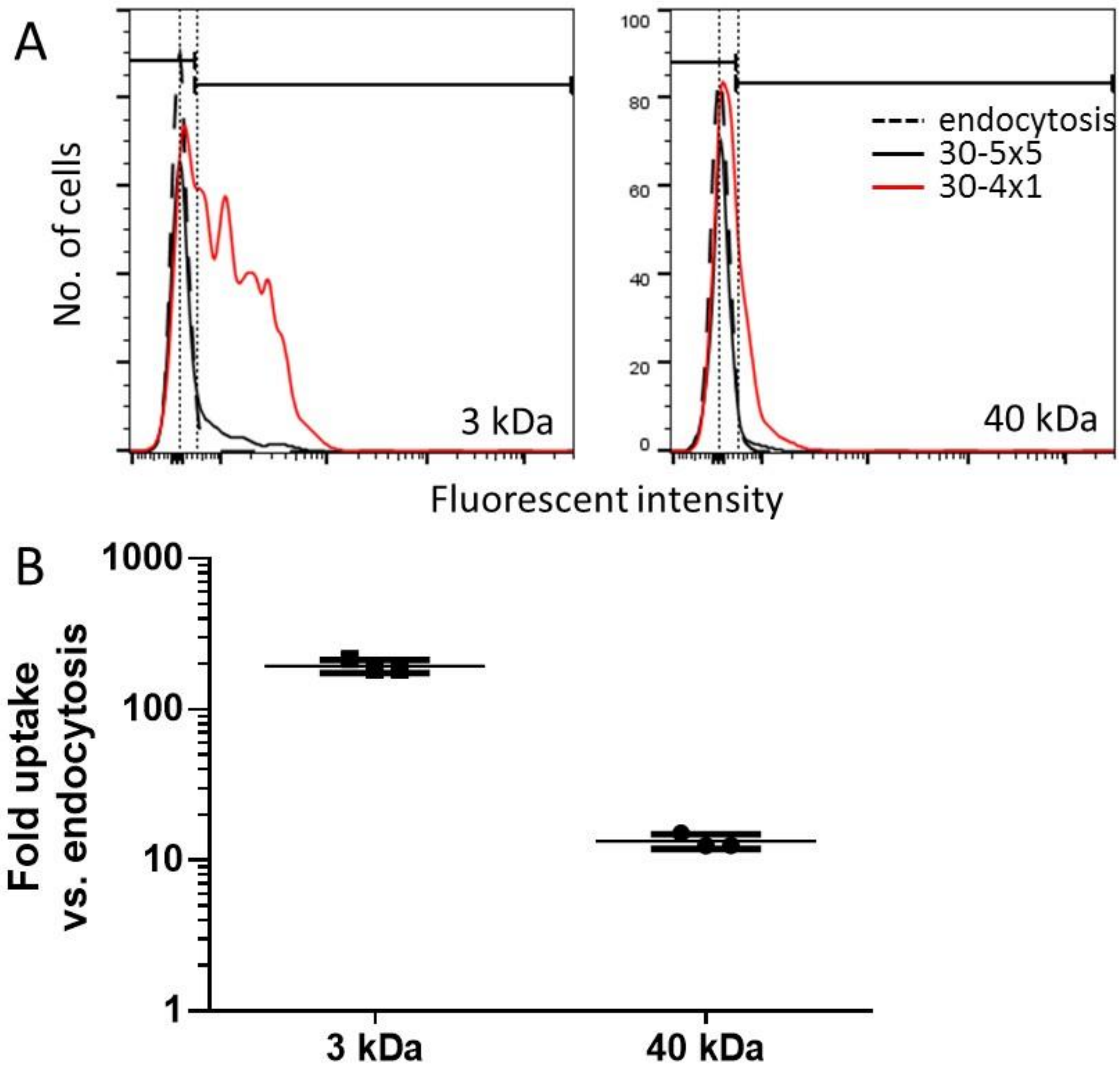

Supplementary Figure S1. Characterization of device design and inter-device performance. A) Representative histograms show the delivery performance using 30-5x5 (black trace) or 30-4x1 (red trace) devices for delivery of 3 and 40 kDa dextrans into polyclonal murine B-cells. **B)** Inter-device performance for 30-4x1 devices is shown. Each point represents performance of a new device (*n*=3 devices in 1 independent experiment, representative of >3 independent experiments). Lines and whiskers represent means ± standard deviation.

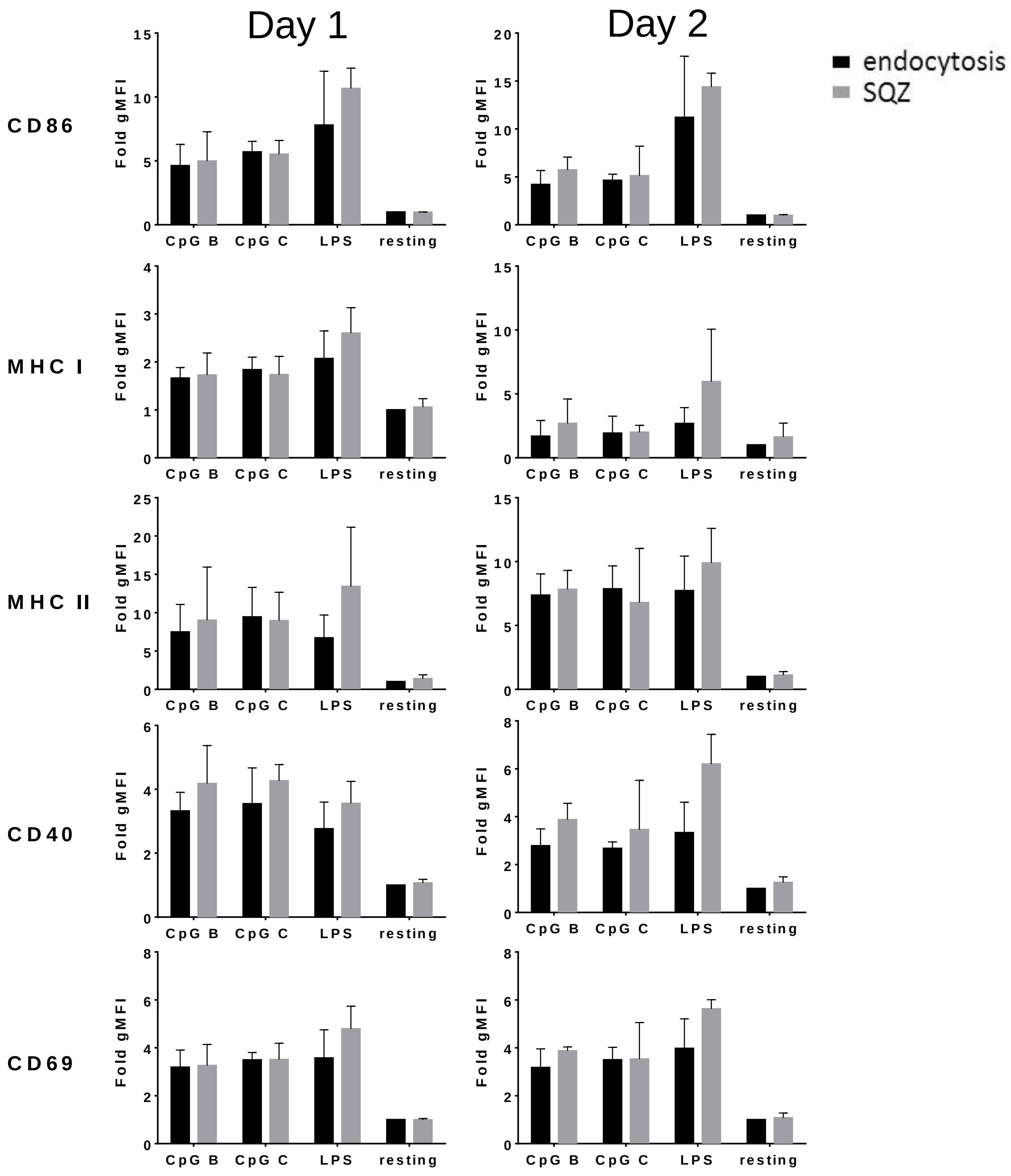

Supplementary Figure S2. Mechano-poration does not affect B-cell activation by diverse stimuli. Quantitative analysis of surface upregulation of multiple markers of B-cell activation and antigen presentation over 2 days for endocytosis and SQZ = squeezed (mechano-porated) B-cells. Flow cytometry was used to measure marker expression and fold geometric mean fluorescent intensity (gMFI) values were calculated by dividing the gMFI of each experimental condition by the average of resting endocytosis B-cells. All data were shown as means ± standard deviation (*n*≥3 independent experiments).

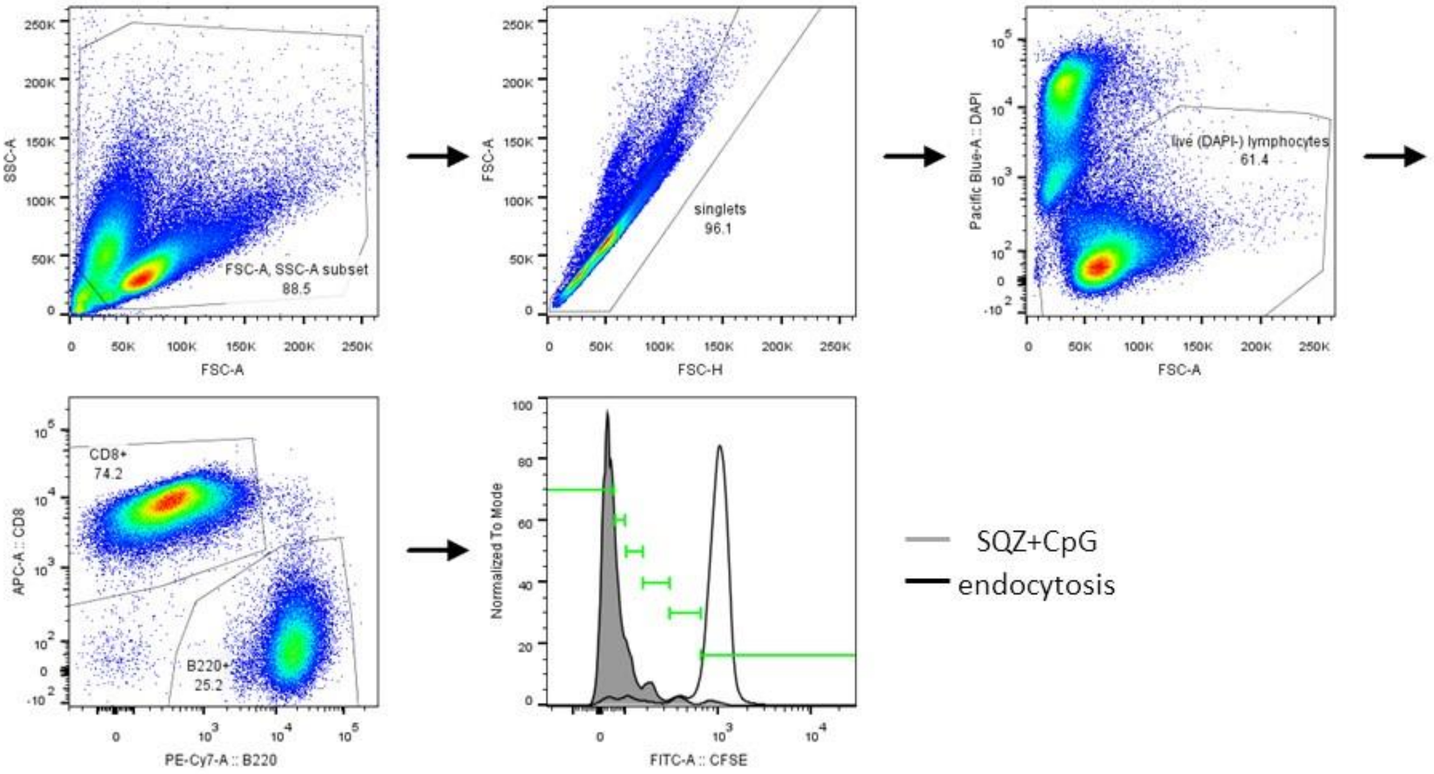

Supplementary Figure S3. Gating strategy for analysing *in vitro* proliferation in B-cell/T-cell co-cultures by flow cytometry. Representative pseudocolor 2D dot plots are shown gating (left to right, top to bottom): cells, singlets, viability ($DAPI^{-}$), and $CD8^{+}$ T-cells and $B220^{+}$ B-cells. $CD8^{+}$ T-cells were then plotted on a histogram and green gates shown were used for calculating generations of cell proliferation overlaid based on CFSE dilution. Representative results were shown overlaid including endocytosis and SQZ+CpG 4 days after co-culture initiation.

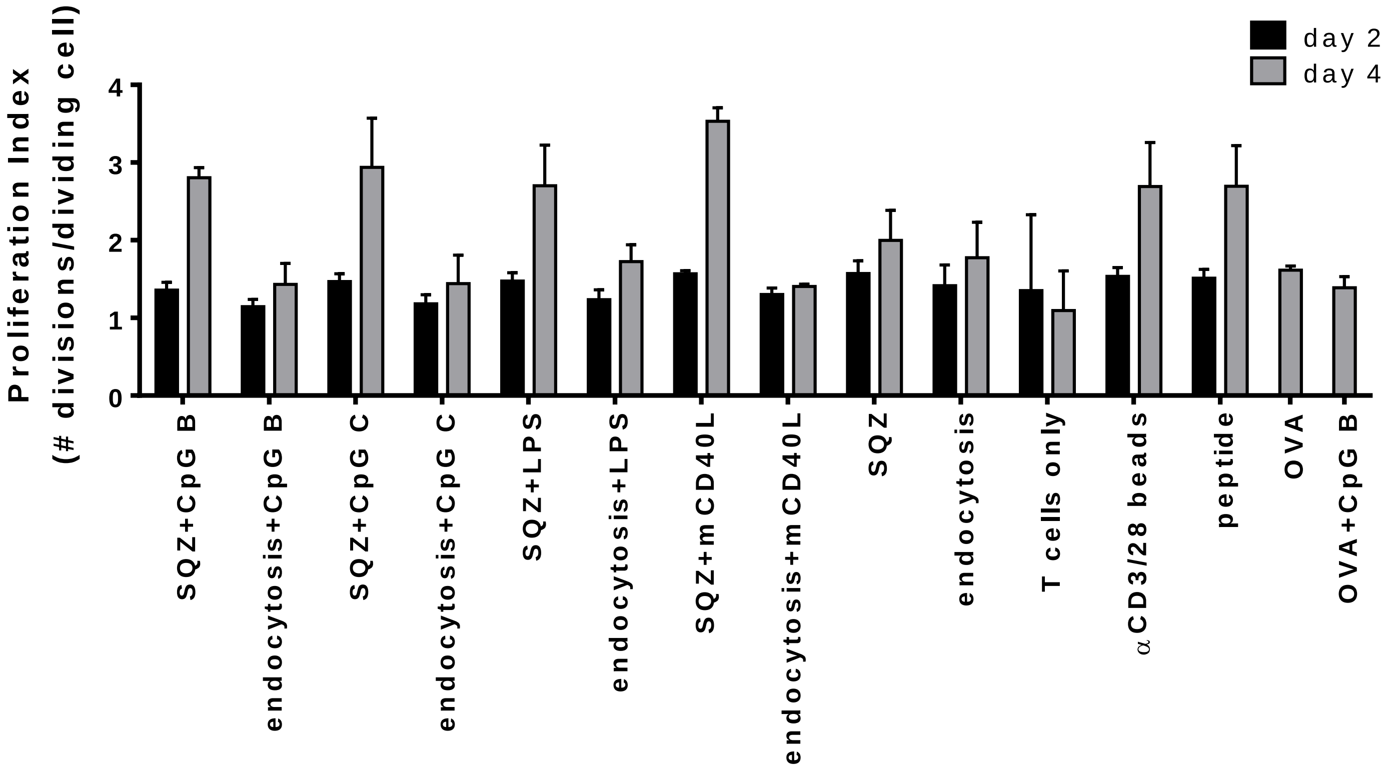

Supplementary Figure S4. Proliferation indices of B-cell/T-cell co-cultures over time. Proliferation indices were calculated as described in Methods. Co-culture stimulation and antigen delivery conditions were shown on the X-axis and the proliferation index on the Y-axis for 2 and 4 days of co-culture (black and grey bars, respectively). All data were shown as means ± standard deviation (*n*≥3 independent experiments).

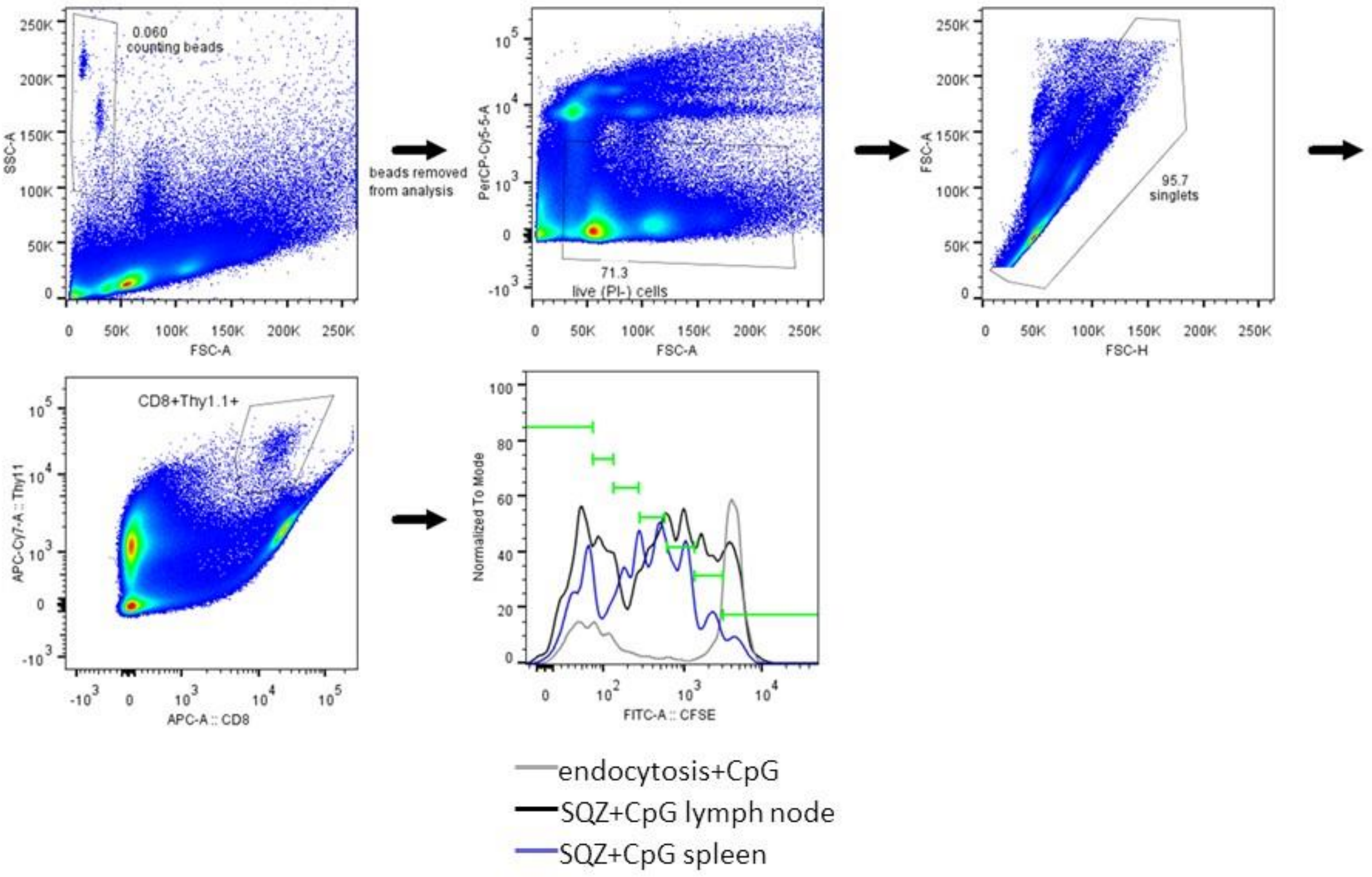

Supplementary Figure S5. Gating strategy for analysing *in vivo* proliferation of adoptively transferred OT-I CD8+ T-cells in spleens and lymph nodes. Representative pseudocolor 2D dot plots are shown gating (left to right, top to bottom): cells, singlets, viability (DAPI/PI$^-$), and OT-I CD8$^+$ Thy1.1$^+$ T-cells. CD8$^+$ T-cells were then plotted on a histogram and green gates shown were used for calculating generations of cell proliferation overlaid based on CFSE dilution. Representative results were shown overlaid including endocytosis+CpG, SQZ+CpG in spleens and in inguinal lymph nodes 4 days after injection of B-cells.
